# Triheptanoin Protects Motor Neurons and Delays the Onset of Motor Symptoms in a Mouse Model of Amyotrophic Lateral Sclerosis

**DOI:** 10.1371/journal.pone.0161816

**Published:** 2016-08-26

**Authors:** Tesfaye W. Tefera, Yide Wong, Mallory E. Barkl-Luke, Shyuan T. Ngo, Nicola K. Thomas, Tanya S. McDonald, Karin Borges

**Affiliations:** 1 School of Biomedical Sciences, The University of Queensland, Brisbane, Queensland, Australia; 2 University of Queensland Centre for Clinical Research, The University of Queensland, Brisbane, Queensland, Australia; University of Florida, UNITED STATES

## Abstract

There is increasing evidence that energy metabolism is disturbed in Amyotrophic Lateral Sclerosis (ALS) patients and animal models. Treatment with triheptanoin, the triglyceride of heptanoate, is a promising approach to provide alternative fuel to improve oxidative phosphorylation and aid ATP generation. Heptanoate can be metabolized to propionyl-CoA, which after carboxylation can produce succinyl-CoA and thereby re-fill the tricarboxylic acid (TCA) cycle (anaplerosis). Here we tested the hypothesis that treatment with triheptanoin prevents motor neuron loss and delays the onset of disease symptoms in female mice overexpressing the mutant human SOD1^G93A^ (hSOD1^G93A^) gene. When oral triheptanoin (35% of caloric content) was initiated at P35, motor neuron loss at 70 days of age was attenuated by 33%. In untreated hSOD1^G93A^ mice, the loss of hind limb grip strength began at 16.7 weeks. Triheptanoin maintained hind limb grip strength for 2.8 weeks longer (p<0.01). Loss of balance on the rotarod and reduction of body weight were delayed by 13 and 11 days respectively (both p<0.01). Improved motor function occurred in parallel with alterations in the expression of genes associated with muscle metabolism. In gastrocnemius muscles, the mRNA levels of pyruvate, 2-oxoglutarate and succinate dehydrogenases and methyl-malonyl mutase were reduced by 24–33% in 10 week old hSOD1^G93A^ mice when compared to wild-type mice, suggesting that TCA cycling in skeletal muscle may be slowed in this ALS mouse model at a stage when muscle strength is still normal. At 25 weeks of age, mRNA levels of succinate dehydrogenases, glutamic pyruvic transaminase 2 and the propionyl carboxylase β subunit were reduced by 69–84% in control, but not in triheptanoin treated hSOD1^G93A^ animals. Taken together, our results suggest that triheptanoin slows motor neuron loss and the onset of motor symptoms in ALS mice by improving TCA cycling.

## Introduction

Amyotrophic Lateral Sclerosis (ALS) is a progressive neurodegenerative disorder characterized by selective degeneration of motor neurons in the brain and spinal cord that leads to muscle weakness, paralysis and death usually due to respiratory failure [1]. The typical age of onset for most forms of ALS is between 50 to 60 years and most patients die 3 to 5 years after symptom onset [2].

Ninety percent of ALS cases are sporadic (SALS), while 10% are familial (FALS) [3]. Mutations in genes such as superoxide dismutase 1 (*SOD1*) [4], TAR DNA-Binding Protein (*TDP 43*) [5], fused in sarcoma (*FUS*) [6], Ubiquilin2 (*UBQLN2*) [7], and *C9ORF72* [8,9] are implicated in genetic causes of ALS. Twenty percent of familial ALS cases are linked to mutations in the SOD1 gene (most commonly occurring mutation in patients with FALS) and this accounts for 1 to 2% of all forms of ALS while FUS and TDP-43 mutations account for 5% of FALS cases [3,10]. SOD1 mutations result in a toxic gain-of-function and are associated with misfolding and mislocalization of the SOD1 protein. While the normal SOD1 protein is usually found in the cytosol, mutant SOD1 accumulates within mitochondria and appears to contribute to many of the mitochondrial perturbations observed in ALS [11–15].

The exact mechanisms underlying selective motor neuron degeneration in ALS are unclear. The mechanisms causing motor neuron loss are multifactorial and may not be mutually exclusive [16]. Among many pathogenic mechanisms, major key pathological processes have been identified including oxidative stress [17], glutamate excitotoxicity [18], inflammation [19], abnormal protein aggregation [4,20], impaired axonal transport [21] and abnormalities in energy metabolism [22,23]. In addition, pathogenic processes in muscle seem to contribute to the progression of disease [24].

### ALS and Energy Metabolism

Mitochondria are the main sites of energy production. The tricarboxylic acid (TCA) cycle together with the electron transport chain produce ATP, the primary cellular energy source that is necessary for cell function and survival. Functional and morphological abnormalities in mitochondria have been shown in the brain, spinal cord and muscles of patients with ALS and in mouse models of ALS [25–35]. Defects in mitochondrial respiration, the electron transport chain as well as ATP synthesis in the spinal cords of hSOD1^G93A^ mice [34], and reduced cellular ATP in the skeletal muscles and cerebral cortex of hSOD1^G93A^ mice have also been observed [36,37]. In this regard, impaired mitochondrial function in ALS is likely to underpin defective energy metabolism and a reduction in the capacity to produce ATP.

Reduced glucose metabolism has been shown in in various brain regions [38–42] and in the spinal cord [37,43] and muscles [36,44] of patients with ALS and mouse models of ALS. Indeed, the expression of several genes that encode for proteins and enzymes involved in glucose uptake, glycolysis, TCA cycle and the electron transport chain were found to be altered in fibroblasts cultured from ALS patients, in the motor cortex of ALS patients, and in the muscle and spinal cords of SOD1 mice [45–49]. Furthermore, there are indications that suggest reduction in levels of TCA cycle intermediates in SOD1 mouse brain and spinal cord and cellular models of ALS [50–52]. Collectively, these findings indicate that impaired ATP production, β-oxidation and TCA cycling contribute to metabolic aberrations in ALS.

**Fig 1:**
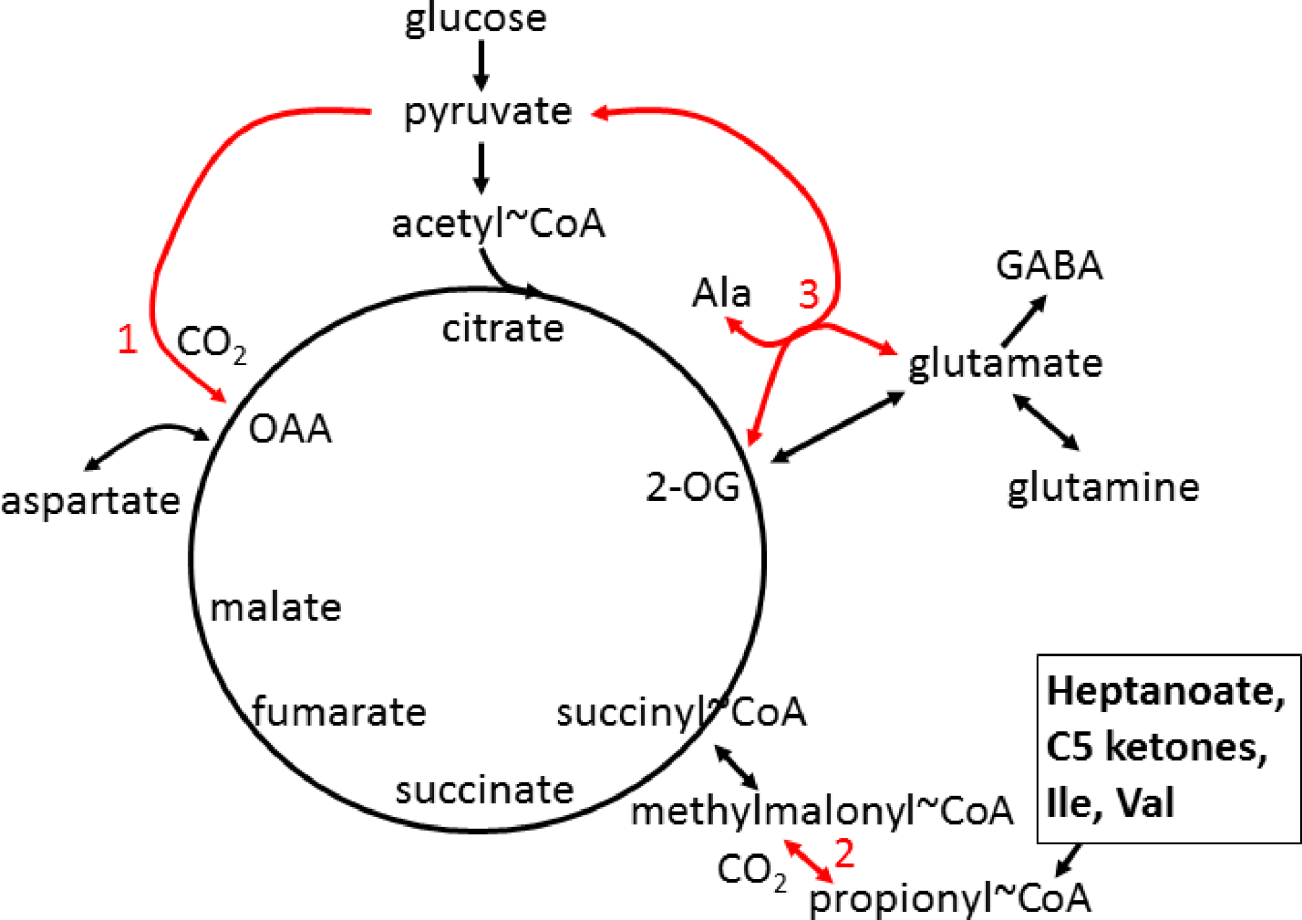
Simplified TCA cycle and anaplerosis in CNS and muscle. Red numbers indicate anaplerotic pathways, that can refill the levels of C4 intermediates of the cycle: **1** pyruvate carboxylase (mostly in CNS), **2** propionyl-CoA carboxylase, and **3** glutamic pyruvic transaminases (very important in muscle). C5 ketones, branched chain amino acids and heptanoate, are metabolized to propionyl-CoA and can therefore be anaplerotic via the propionyl-CoA carboxylation pathway. OAA–oxaloacetate, 2-OG– 2-oxoglutarate, Ile–isoleucine, Val—valine.

Reduced levels of aspartate, glutamine and GABA in the spinal cord of hSOD1^G93A^ mice during early disease stages suggest that C4 intermediate levels of the TCA cycle are decreased [51]. In the CNS, glutamine supplies C4 carbons to glutamate and subsequently GABA and 2-oxoglutarate (α-ketoglutarate), while aspartate can be deaminated to form oxaloacetate (Fig 1). Low levels of oxaloacetate that can occur due to low supply of C4 TCA cycle intermediates and metabolites, including aspartate and glutamine, may impair the entry of acetyl-CoA generated from fuels into the TCA cycle. Therefore, the use of an alternative and additional fuel source that can “re-fill” the C4 carbon deficient TCA cycle intermediates (anaplerosis) may be of benefit in alleviating metabolic defects in ALS. When needed, anaplerosis is likely to improve ATP production in both the CNS and muscle especially during periods of energetic stress [53]. Thus, we suggest anaplerosis as a potential treatment approach for ALS.

### Anaplerosis and Triheptanoin as a Treatment Approach

Anaplerosis serves to “re-fill” the levels of C4 carbon-deficient TCA cycle intermediates to improve energy supply during periods of increased energy need [53]. Anaplerotic enzymes include pyruvate carboxylase (*Pcx*), which produces oxaloacetate and glutamic pyruvic transaminases 1 and 2 (G*pt1* and *2*), which catalyze the reaction pyruvate + glutamate < = > α-ketoglutarate + alanine (Fig 1).

Triheptanoin, the triglyceride of heptanoic (C7) acid, not only provides an alternative fuel source in the form of medium chain fats, that are typically absent in a standard normal diet, but it can also be anaplerotic. It is a novel metabolic therapeutic that is being used in clinical pilot studies to treat patients with various disorders associated with metabolic dysfunction. This includes genetic metabolic disorders of fatty acid oxidation [53,54] and neurological and muscular disorders [55–57]. Triheptanoin provides the body with heptanoate, which diffuses into the mitochondria of cells in both the CNS and peripheral tissues to be metabolized to acetyl-CoA and propionyl-CoA by β-oxidation. Also, the liver converts heptanoate into the “C5 ketones”, β-hydroxypentanoate and β-ketopentanoate, which are then released into blood. After being taken up via monocarboxylate transporters into cells of various tissues, including the CNS, “C5 ketones” are also β-oxidized to acetyl-CoA and propionyl-CoA. Carboxylation of propionyl-CoA produces succinyl-CoA, a C4 TCA cycle intermediate via propionyl-CoA carboxylation, an anaplerotic pathway that has been described in various tissues [58–60] (Fig 1). An adequate supply of C4 TCA cycle intermediates is important for optimal oxidation of fuels by the TCA cycle [53,61].

There is now increasing evidence that triheptanoin improves energy metabolism in the CNS and muscle of patients and in animal models of different diseases, including brain and muscle in Huntington’s Disease patients [55,62] and models of epilepsy [60,63]. Triheptanoin also was found to provide alternative fuel to the brains of patients and mice with glucose transporter 1 deficiency, which show impaired glucose uptake into the CNS [56,59]. In addition, there is evidence that triheptanoin can prevent cell loss in the brain in mouse models of stroke [64] and Canavan disease [65] by improving mitochondrial respiration.

Despite the beneficial effects observed following triheptanoin treatment in multiple models of disease and neurodegeneration, the use of triheptanoin as a potential therapeutic in ALS remains unexplored. Thus, we used hSOD1^G93A^ mice to test the hypotheses that 1) triheptanoin attenuates motor neuron death and delays the onset of motor symptoms, and that 2) the expression of enzymes involved in energy metabolism is diminished in muscle and can be rescued by triheptanoin.

## Materials and Methods

### Animals

All experiments were approved by the University of Queensland Animal Ethics Committee and followed the guidelines of the Queensland Animal Care and Protection Act 2001. Wild-type and hSOD1^G93A^ mice (B6.Cg-Tg(SOD1*G93A)1Gur/J, stock no. 004435, Jackson laboratory, Bar Harbor, ME, USA), were generated by mating hSOD1^G93A^ males with C57BL/6 wild-type females (University of Queensland). Mice were housed in a 12 hour light, 12 hour dark cycle, and had free access to food and water. Experimenters were blinded to animal genotype (until the mice started expressing the ALS phenotype) and treatment.

### Triheptanoin Treatment

Starting at P35, female wild-type and hSOD1^G93A^ mice were given either control or triheptanoin-containing diet treatment until they were sacrificed. Untreated mice received SF11-027, a diet with ingredients typical to other mouse diets (Specialty Feeds, Glen Forrest, WA, AUS). Treated wild-type and hSOD1^G93A^ mice received a matched formulation (SF11-028, Specialty Feeds) in which 35% of the calories were from triheptanoin. This dose was chosen, as it is also used in the clinic to treat patients with metabolic disorders and has shown beneficial metabolic and protective effects in several animal models of various disorders [55,56,60,62–64,66]. The diets were matched in protein, mineral, antioxidant and vitamin content relative to their caloric densities [66]. Triheptanoin replaced sucrose, some of the complex carbohydrates and long chain fats. For the motor neuron count experiments, triheptanoin was provided by Ultragenyx Pharmaceutical Inc. (Novato, CA, USA). All other experiments were performed with triheptanoin obtained from Sasol GmbH (Germany).

### Motor Neuron Counts

Mice were deeply anesthetized with pentobarbital (120 mg/Kg i.p. Provet, Northgate, QLD, AUS) and euthanized by decapitation. Spinal cords were flushed out of the spinal cavity using a cold phosphate buffered saline (PBS) filled syringe that was fitted with a blunted 23-gauge needle. Spinal cords were immediately fixed in 4% paraformaldehyde (PFA, pH 7.4) for 24 to 48 hours and then embedded in liquid paraffin. Serial, transverse sections (16μm) were obtained using a Leica Rotary Microtome. Motor neurons were identified after staining with thionine (0.1%) in acetate buffer (pH = 3.9). The L4 to L5 regions were identified according to standard anatomical guidelines described in [67] using an Olympus BX61 upright light microscope with 10x, 20x and 40x objectives. Briefly, the L4 and L5 sections were identified according to the area of the gracile fasciculus and dorsal corticospinal tract relative to the spinal cord central canal. In L3 sections, the dorsal corticospinal tract is smaller than in L4 and L5. Motor neuron numbers in L4 and L5 were determined using stereological principles based on the counting of every 10^th^ section with a total of 11 to 16 sections counted [68]. Every tenth consecutive L4 and L5 spinal cord section was counted until the sacral dorsal commissural nucleus appeared concomitant with a decrease in size of the gracile fasciculus and dorsal corticospinal tract, which are markers for L6. Motor neurons were identified by a large, darkly stained cell body, a pale nucleus with a continuous boundary and one or more darkly stained nucleoli [67].

### Blood ketone and glucose measurements

Blood plasma was collected in EDTA-coated tubes from the mice used for motor neuron counting, centrifuged at 2,000g for 10 mins. It was then stored at -80°C until analysis. Plasma levels of the ketone β-hydroxybutyrate and glucose were determined using Cayman colorimetric assay kits (Ann Arbor, MI, USA), according to the manufacturer’s instructions.

### Behavioral Testing and Observation

Animals underwent behavioral testing approximately 3–4 hours into the light cycle. All behavioral testing was conducted in an environment with minimal stimuli so as to minimize any possible effects caused by changes in external stimuli. Animals were weighed before every test session. Mice were observed, and disease progression tracked and graded according to a neurological score sheet [69] to ensure that any non-ALS related deaths were excluded from the study. In accordance with ethical requirements, hSOD1^G93A^ mice that became too weak to reach the food hoppers were provided with wet chow on the floor of the cages. The endpoint of the study was defined as the mouse being unable to right itself within 15 seconds after being placed on its back. Upon reaching end-stage or 25 weeks of age, transgenic mice and their respective wild-type littermates were euthanized with pentobarbital (120 mg/kg, i.p., Provet). Tissues, including the gastrocnemius muscle, were collected for subsequent analysis. To measure the time point when body weight loss started, we defined the day where a loss of more than 10% in an individual mouse occurred relative to its mean body weight from week 12 to 17. Also all subsequent three body weight measurements were ≤ 90% of the original mean weight.

### Hind Limb Grip Strength Test

Hind limb grip strength tests were conducted twice a week using a T-bar force transducer (Ugo Basile, Varese, ITA). The animal was held by the tail, ensuring its hind limbs were gripping the T-bar before being pulled downwards at a 60° angle. The reading on the force transducer was taken only if both hind limbs released the bar at the same time. The average of 10 trials per mouse was recorded for each training session [70]. To compare time points of grip strength loss, we determined the time point where a grip strength loss of more than 30% occurred in an inidividual mouse relative to its mean grip strength of week 9 to 13 and the subsequent three measurements were ≤ 70% of the original average strength.

### Rotarod Test

Rotarod tests were conducted with 10 repeats once a week using a rotarod designed for mice (Ugo Basile). Animals were placed on the rod, which was then rotated for 3 min at 25 revolutions per minute [70]. The time at which the animal fell off was recorded. We defined the age of balance loss on the rotarod when this time was zero.

### RNA Extraction, cDNA Synthesis, Quantitative Real Time PCR Assay

After euthanasia, the gastrocnemius muscle was quickly removed and frozen in liquid nitrogen. To extract RNA, muscle samples were pulverized in liquid nitrogen with a cold mortar and pestle, dissolved with TRI reagent (Life Technologies, Carlsbad, CA, USA) and extracted according to the manufacturer’s instructions. Contaminating DNA was removed by DNAse I treatment and cDNA was synthesized using the Tetro cDNA synthesis kit (Bioline, London, UK) according to the manufacturer’s instructions.

The expression of several metabolic genes was assayed (Table 1) by quantitative real time PCR.

**Table 1:** Gene names, symbol, forward and reverse primer sequences and of primers used for the gene expression studies of metabolic genes.

Gene	Symbol	Sequence 5’ to 3’
β2-microglobulin	*B2m*	F AGACTGATACATACGCCTGCR ATCACATGTCTCGATCCCAG
Hydroxymethylbilane synthase	*Hmbs*	F AAGGGCTTTTCTGAGGCACCR AGTTGCCCATCTTTCATCACTG
TATA binding protein	*Tbp*	F TTCTCGAAAGAATTGCGCTGTR GCCTTGTGAGTCATTTCAGTG
Propionyl-CoA Carboxylase (Subunit α)	*Pcca*	F AGAATTGCAAGGGAAATTGG R CTAAAGCCATCCCTGGTCTC
Propionyl-CoA Carboxylase (Subunit β)	*Pccb*	F AGCCTACAACATGCTGGACA R GGTCCTCCCATTCATTCTTG
Methylmalonyl-CoA mutase	*Mut*	F CCAAACACTGACCGTTCTCA R GGAATGTTTAGCTGCTTCAGG
Pyruvate carboxylase	*Pcx*	F GAGCTTATCCCGAACATCCC R TCCATACCATTCTCTTTGGCC
Pyruvate dehydrogenase E1 α 1	*Pdha1*	F AACTTCTATGGAGGCAACGG R CTGACCCTGATTAGCAGCAC
Glyceraldehyde-3-phosphate dehydrogenase	*Gapdh*	F ATACGGCTACAGCAACAGGG R TCTTGCTCAGTGTCCTTGCT
Oxoglutarate dehydrogenase	*Ogdh*	F TGCAGATGTGCAATGATGAC R GCAGCACATGGAAGAAGTTG
Succinate dehydrogenase complex (Subunit A)	*Sdha*	F GGAACACTCCAAAAACAGACCT R CCACCACTGGGTATTGAGTAGAA
Glutamate-pyruvate transaminase 1	*Gpt1*	F TGAGGTTATCCGTGCCAATAR GTCCGGACTGCTCAGAAGAT
Glutamate-pyruvate transaminase 2 (alanine aminotransferase)	*Gpt2*	F GCGACGGTATTTCTACAATCCR CGCGGAGTACAAGGGATACT

All primer pairs were evaluated for efficiency using a 4 fold serial dilution series of muscle cDNA. The derived slope of each primer pair was used to calculate the efficiency by applying the formula, 4^[(-1/slope)-1]*100^. Reactions consisting of diluted cDNA, 5μl of SYBR Green master mix and 8μM of forward and reverse primers were amplified after an initial hot start. The thermal profile for the assay was an initial hot start of 95°C for 10 minutes, followed by 40 cycles of 95°C for 30 seconds, 60°C for 1 minute and 72°C for 30 seconds (ABI 7900HT Fast Real-Time PCR system, Applied Biosystems). Lastly the melt curve was generated by heating to 95°C for 2 minutes, cooling to 60°C for 15 seconds and a final 2% heating ramp to 95°C for 15 seconds. Samples without reverse transcriptase treatment were included to ensure that samples were free from DNA contamination. To select reference genes for normalization, expression of ten housekeeping genes in the different experimental groups were analyzed using GeNorm function. TATA binding protein (*Tbp*),β2-microglobulin (*B2m*) and Hydroxymethylbilane synthase (*Hmbs*) were chosen because they were the least changed in the experimental groups. The fold expression (ΔCT) of the gene of interest (goi) relative to the geometric mean of housekeeping genes (*Tbp*, *B2m* and *Hmbs*) were calculated with a formula adapted from [71] taking into consideration the individual efficiencies (E) of each primer pair.

$${\Delta CT}_{goi}\;=\;2^{-}\left[{(CT}_{goi}{Log}_{2}E_{goi}\right)-3\sqrt{\left({CT}_{TBP}{Log}_{2}2.03\right)\left({CT}_{B2m}{Log}_{2}2.03\right)\left({CT}_{HMBS}{Log}_{2}1.86\right)}]$$

### Enzyme Assay

Mitochondrial extracts were prepared from the gastrocnemius muscle of male wild-type and hSOD1^G93A^ mice at different disease stages and homogenized with a glass-teflon homogenizer in 500 μL ice cold extraction buffer (0.32 M sucrose, 1 mM EDTA and 10 mM Tris-HCl, pH 7.4). Samples were centrifuged at 1000 g for 10 min at 4°C and the supernatant was then centrifuged at 12,000 g for 15 min at 4°C. The pellet was washed in 500 μL extraction buffer followed by another centrifugation at 12,000g for 15 min and then resuspended in cold extraction buffer with 0.1% Triton X-100. The ratio of buffer to tissue was 2.5 mL/g.

The maximal activities of 2-oxoglutarate dehydrogenase in these enriched mitochondrial extracts were determined via the reduction of nicotinamide adenine dinucleotide (β-NAD^+^) in buffer (75 mM Tris HCl (pH 8), 1 mM ethylenediaminetetraacetic acid, 0.5 mM thiamine pyrophosphate, 1.5 mM Coenzyme A, 4 mM β-NAD^+^, 1mM DTT, 2 mM calcium chloride) initiated with 15 mM 2-oxoglutarate, with background activity measured when no substrate was added [72]. The rate of NAD^+^ reduction was measured with a spectrophotometric plate reader (Tecan, Mannedorf, CH), background activity was subtracted and the resultant activity was normalized to protein content measured with a Pierce Bicinchoninic acid assay (ThermoFisher Scientific, Scoresby, VIC, AUS). The amount of protein used was 10 μg per well.

### Data Analysis

Statistical analyses were performed with Graphpad Prism (versions 5.03 and 6.0) using two-way ANOVAs followed by Bonferroni multiple comparisons post-hoc tests for analysis of several groups. For the comparison of the onset of body weight loss and the area under the curve (AUC) for hind limb grip strength, two-tailed unpaired t-tests were employed. Data are represented as mean ± SEM. Using the variation of our motor neuron numbers in our colony determined in a preliminary experiment, power analysis showed that to be able to observe a treatment effect of 30% with α = 0.05 and β = 80%, n = 8–9 mice were necessary, an sample size which is higher than in the guidelines [73]. In addition, power analysis using the average standard deviations for the onset of loss of grip strength and balance on rotarod showed that n = 5 was sufficient to observe changes between means by 2.3 and 1.5 weeks respectively with 80% power at the 0.05 significance level. We are aware that our study was underpowered for survival analyses based on the guidelines for preclinical ALS research [73].

## Results

### Triheptanoin Protected Against Motor Neuron Loss in hSOD1^G93A^ Mice

**Fig 2:**
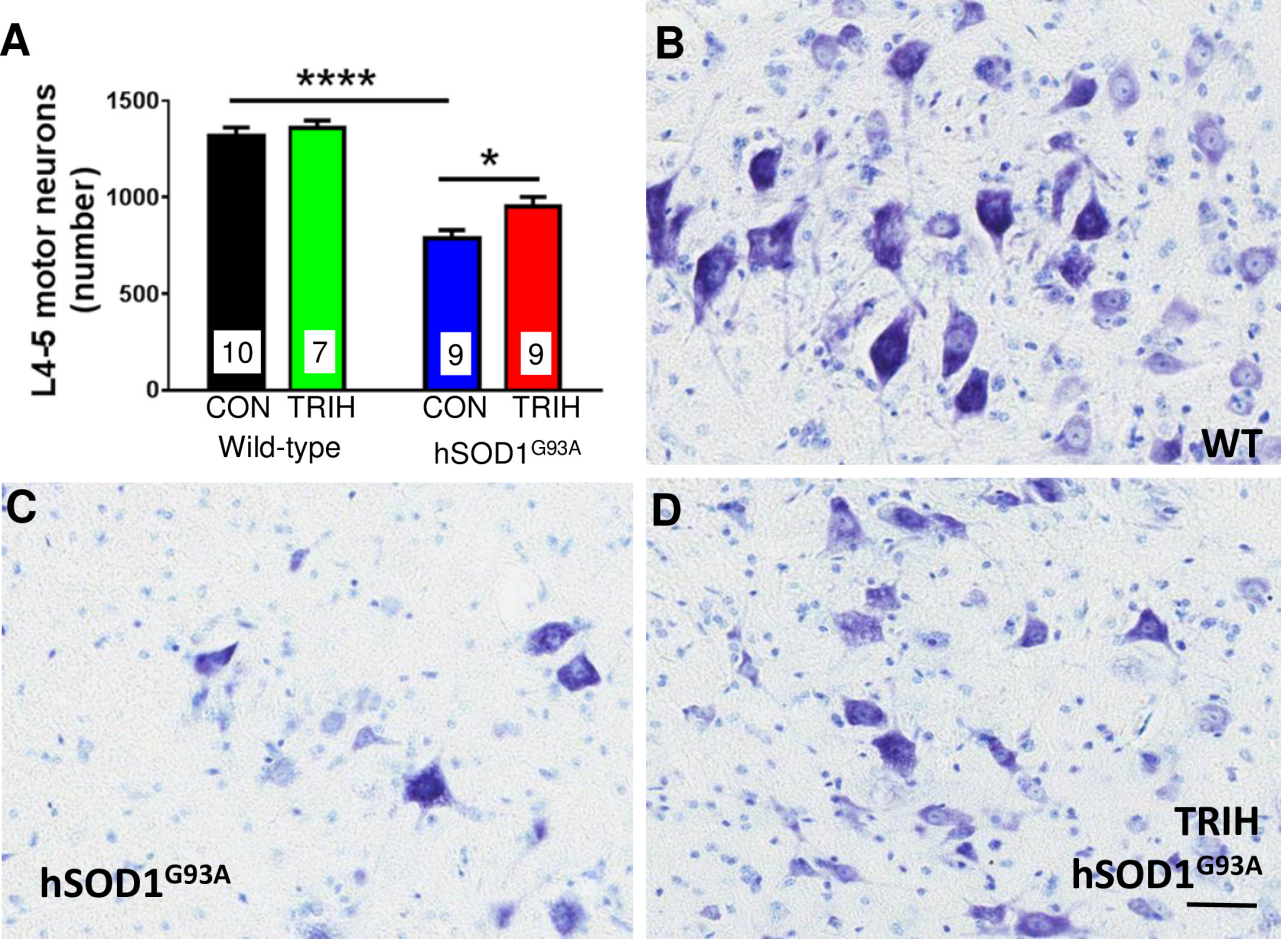
Triheptanoin preserves motor neurons. Starting at P35, female wild-type and hSOD1^G93A^ mice were either treated with triheptanoin (TRIH) or control (CON) diet until P70. (A) Stereologically counted motor neuron numbers in the L4-L5 segments of these 70 day old mice (n = 7–10) revealed a 38% loss of motor neurons in control-fed hSOD1^G93A^ mice. Triheptanoin provided a 33% protection against motor neuron loss. Two way ANOVA p<0.0001 for genotype, p = 0.0126 for treatment, the stars indicate results from a Bonferroni multiple comparisons post hoc tests if significant (**** p<0.0001, * p<0.05) (B-D) Representative thionine stained spinal cord sections from an untreated wild-type mouse (B) and untreated (C) and triheptanoin-treated (D) hSOD1^G93A^ mice with arrows pointing to the motor neurons counted. Scale bar 100 μm.

A defining hallmark of ALS is progressive motor neuron loss. Here, we confirmed that relative to wild-type mice, 38% of lumbar L4-L5 motor neurons were lost by 70 days of age in hSOD1^G93A^ mice (Fig 2, two-way ANOVA p<0.0001 for genotype, Bonferroni multiple comparisons post hoc test p<0.0001, n = 7–10 per group). To determine if triheptanoin can protect motor neurons, we initiated triheptanoin treatment at P35 in female hSOD1^G93A^ mice at 35% of caloric intake until P70. This dose was chosen, as it is also used in the clinic to treat patients with metabolic disorders and has shown beneficial metabolic and protective effects in several animal models of various disorders [55,56,60,62–64,66]. Starting triheptanoin treatment at P35 in hSOD1^G93A^ mice resulted in higher motor neuron numbers by 18% relative to untreated hSOD1^G93A^ mice (two-way ANOVA p = 0.0126 for treatment, Bonferroni multiple comparisons post hoc test p<0.05), amounting to a 33% protection against motor neuron loss.

### Triheptanoin Delayed the Onset of the Loss of Hind Limb Grip Strength and Balance in hSOD1^G93A^ Mice

**Fig 3:**
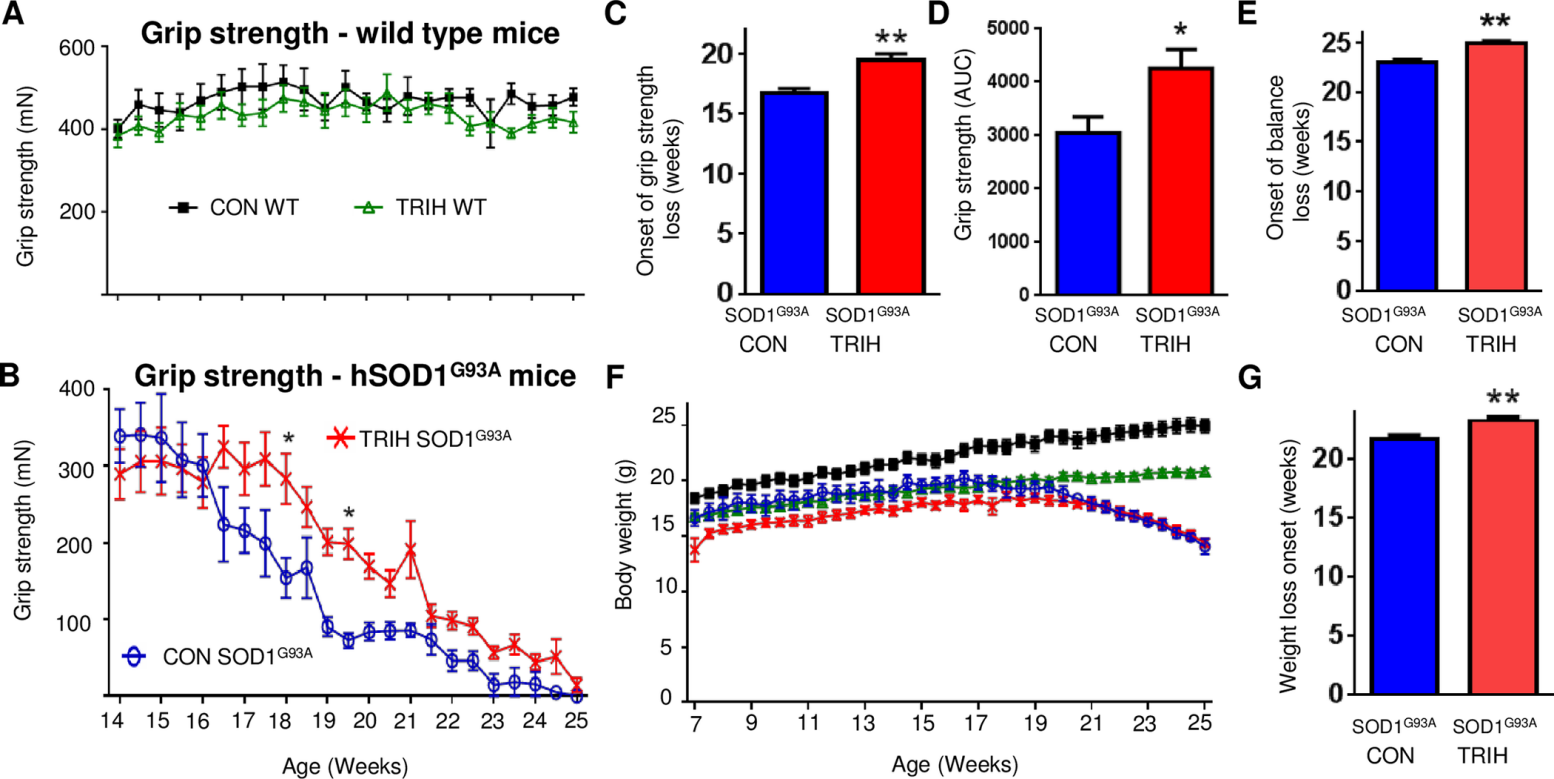
Triheptanoin treatment delays the loss of hind limb grip strength in hSOD1^G93A^ mice. Starting at P35, female wild-type and hSOD1^G93A^ mice were treated with triheptanoin (TRIH) or control (CON) diet until they were sacrificed. (A) No differences in grip strength was observed between triheptanoin (green open triangles, n = 15) and control treated wild-type mice (black filled squares, n = 12). (B) The grip strength over time differed in triheptanoin treated (red crosses, n = 8) vs. untreated (blue empty circles, n = 5) hSOD1^G93A^ mice (p = 0.04, two way ANOVA), with treated mice having higher grip strength at 18 and 19.5 weeks (p<0.05 Bonferroni post-hoc test). (C) The onset of hind limb grip strength loss was delayed by 2.8 weeks in triheptanoin treated hSOD1^G93A^ mice when compared to untreated hSOD1^G93A^ mice (p = 0.002, t-test). (D) Overall hind limb grip strength shown as the area under the curve over time was increased in triheptanoin treated hSOD1^G93A^ mice compared to control treated hSOD1^G93A^ mice (p = 0.02, t-test). (E) The onset of balance loss in triheptanoin treated hSOD1^G93A^ mice was significantly delayed by 13 days (p = 0.0016, t-test). (F) Body weights over time were significantly different between triheptanoin treated vs. untreated wild-type mice and treated vs. untreated hSOD1^G93A^ mice. (G) The onset of body weight loss in triheptanoin treated vs. untreated hSOD1^G93A^ mice (n = 5) was delayed (p = 0.007, t-test). * p<0.05, ** p<0.01. The onset of body weight loss was defined as the day where a loss of more than 10% in an individual mouse occurred relative to the mean body weight from week 12 to 17 was observed. Also all subsequent three body weight measurements were ≤ 90% of the original mean weight.

We investigated the extent to which triheptanoin delays the onset of the progressive loss of muscle strength in hSOD1^G93A^ mice. Hind limb grip strength tests were used to assess the course of disease progression in treated vs. untreated mice. There was no observable difference between the mean hind limb grip strength of the treated (n = 15) vs. untreated (n = 12) wild-type mice. Mean hind limb grip strength for both groups of wild-type mice consistently fell between 300 and 600 mN (Fig 3A). The hind limb grip strength of hSOD1^G93A^ mice on both treatments never exceeded 400 mN (Fig 3B). The time at which reduced hind limb grip strength became apparent was significantly later in triheptanoin treated hSOD1^G93A^ mice when compared to untreated hSOD1^G93A^ mice (n = 8 for triheptanoin treated, n = 5 for untreated mice; Fig 3B, p = 0.04, two way ANOVA). Bonferroni’s multiple comparisons tests indicated that at 18 and 19.5 weeks of age, triheptanoin treated hSOD1^G93A^ mice had higher hind limb grip strength when compared to untreated hSOD1^G93A^ mice (Fig 3B, p<0.05). Untreated hSOD1^G93A^ mice began to lose hind limb grip strength at 16.7 weeks of age, but the time of onset of the loss of hind limb grip strength was delayed by 2.8 weeks in hSOD1^G93A^ mice that received oral triheptanoin (p = 0.002, Fig 3C). The area under the curve for the grip strength over time for each mouse treated with triheptanoin was increased by 38% (p = 0.024, t-test, Fig 3D).

In the rotarod test, behavior of the hSOD1^G93A^ mice varied widely. Many mice seemed “unmotivated” to perform on the rotating rod and no satisfying rotarod baseline performance was reached. Therefore we could only assess the time point when mice were unable to stay on the rod. Triheptanoin treatment delayed the time of onset of loss of balance on the rod by 13 days (p = 0.0016; Fig 3E).

### Body Weight Loss and Survival in hSOD1^G93A^ Mice with and without Triheptanoin

Body weight is another indicator of disease progression in hSOD1^G93A^ mice. When compared to untreated wild-type mice (n = 12), wild-type mice on oral triheptanoin (n = 15) gained weight at a slower rate (p<0.0001, Fig 3F). At 14 weeks of age, triheptanoin treated wild-type mice were approximately 3g lighter than untreated wild-type mice (p<0.05). This weight difference increased to approximately 4g at 22 weeks of age.

Both treated and untreated hSOD1^G93A^ mice (n = 5–8) were lighter when compared to untreated wild-type mice (p<0.0001). There were no differences in weight between hSOD1^G93A^ mice with or without triheptanoin treatment over the full time period. However, compared to control diet, triheptanoin fed hSOD1^G93A^ mice showed a trend of reduced body weight gain from 7–16 weeks. After 20 weeks of age, the body weight of hSOD1^G93A^ mice on the control and triheptanoin diet became similar (Fig 3F). The onset of body weight loss in hSOD1^G93A^ mice was delayed by 1.6 weeks (11 days) in triheptanoin treated mice (Fig 3G, p = 0.007, unpaired two-tailed t-test).

Based on power analysis and the guidelines for ALS research [73], this study of motor symptoms was too small to assess survival with adequate power. No differences were seen in the number of days taken to reach end-stage when comparing triheptanoin treated (n = 7) to untreated (n = 5) hSOD1^G93A^ mice (174.9±3.5 vs. 172.4±3.9, p = 0.653), indicating that in this small study, triheptanoin treatment did not alter survival.

### Expression and Enzyme Activity Studies

**Fig 4:**
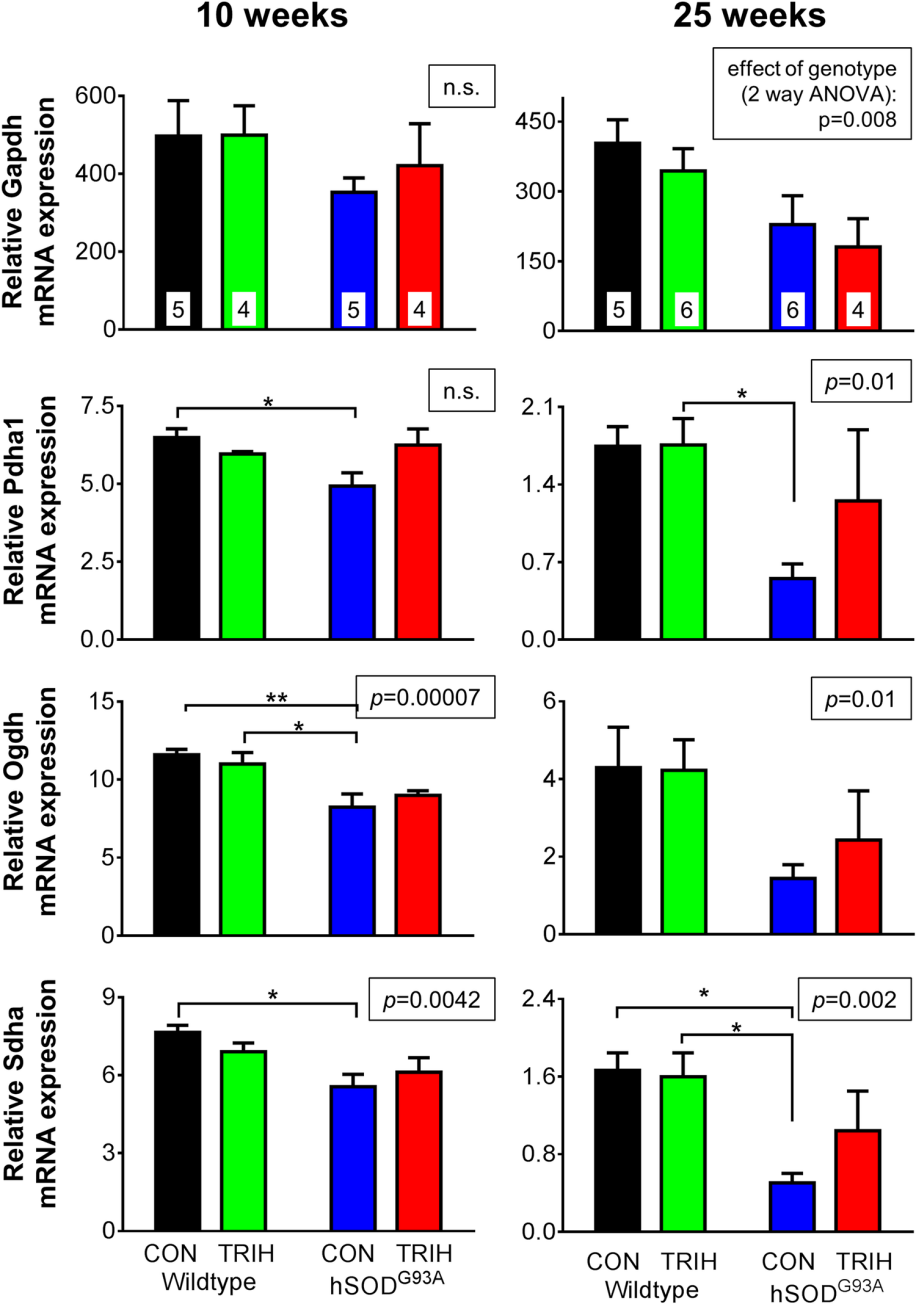
*Gapdh*, *Pdha*, *Ogdh* and *Sdha* mRNA expression in gastrocnemius muscle after triheptanoin treatment. Starting at P35, female wild-type and hSOD1^G93A^ mice were treated with triheptanoin (TRIH) or control (CON) diet until they were sacrificed. Quantitative real time PCR analysis of *Gapdh*, *Pdha1*, *Ogdh* and *Sdha* of the gastrocnemius muscle of 10 and 25 week old wild-type and hSOD1^G93A^ mice untreated or treated with triheptanoin relative to house keeping genes. N-numbers of each group used in all graphs are indicated in the top bar graphs. The insets above each graph show the p-values for the effects of genotype in two-way ANOVAs, while the effect of diet was p>0.05 for each bar graph. When significant, the results of Bonferroni post tests are shown by stars (* p<0.05, ** p<0.01), showing that decreases of mRNA levels of several enzymes in hSOD1^G93A^ mice were not apparent with triheptanoin treatment.

**Fig 5:**
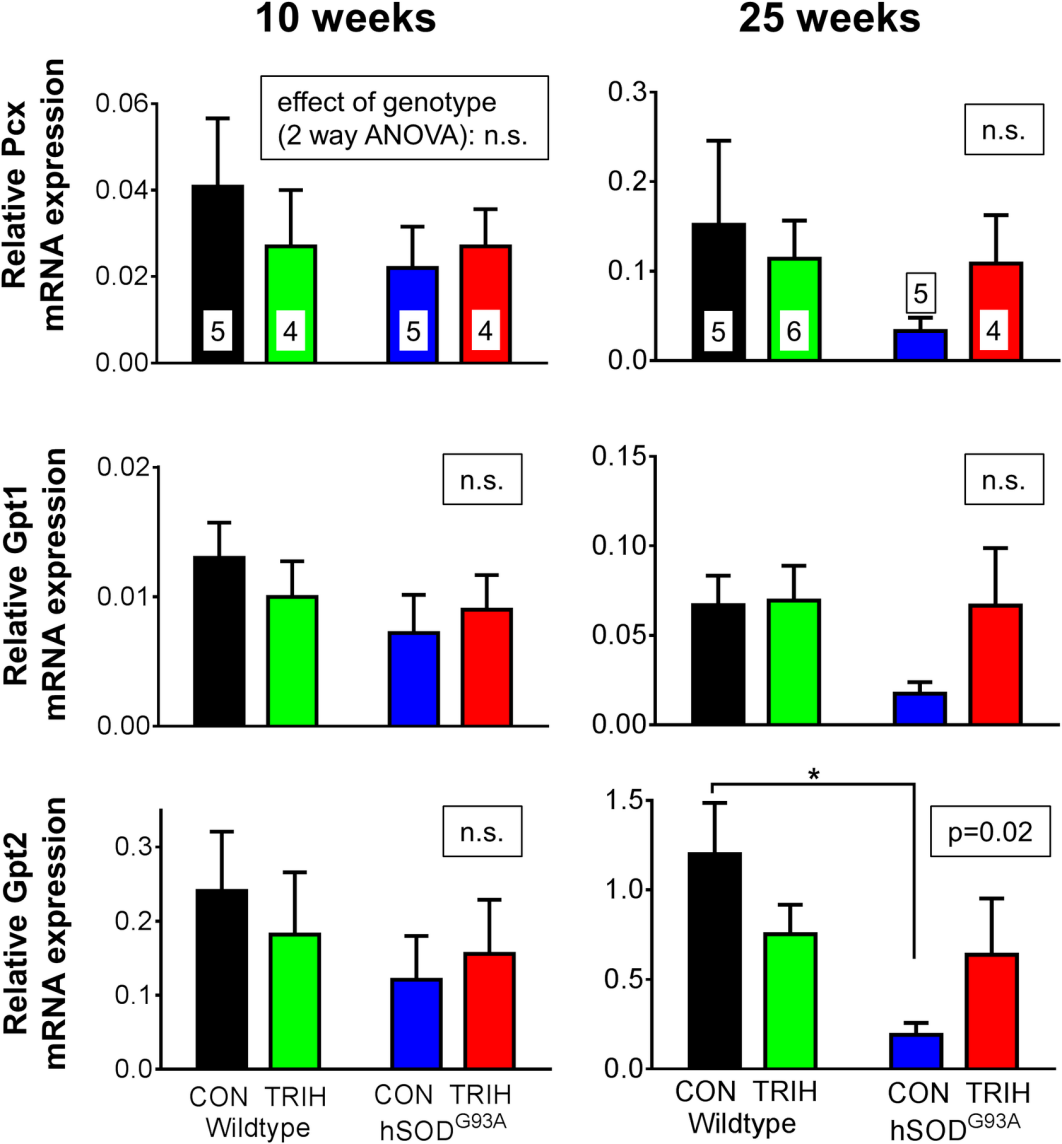
Relative expression of the anaplerotic genes, pyruvate carboxylase *Pcx* and glutamic pyruvic transferases *Gpt1* and *2*. Starting at P35, female wild-type and hSOD1^G93A^ mice were either treated with triheptanoin (TRIH) or control (CON) diet until they were sacrificed. Gene expresssion is compared in the gastrocnemius muscle of 10 and 25 week old triheptanoin treated vs. untreated wild-type and hSOD1^G93A^ mice relative to housekeeping genes. N-numbers of each group used in all graphs are indicated in the top bar graphs. The insets above each graph show the p-values for the effects of genotype in two-way ANOVAs, while the effect of diet was p>0.05 for each bar graph. When significant, the results of Bonferroni post tests are shown by a star (* p<0.05), indicating that triheptanoin treatment prevented the decrease of expression in *Gpt2* mRNA.

**Fig 6:**
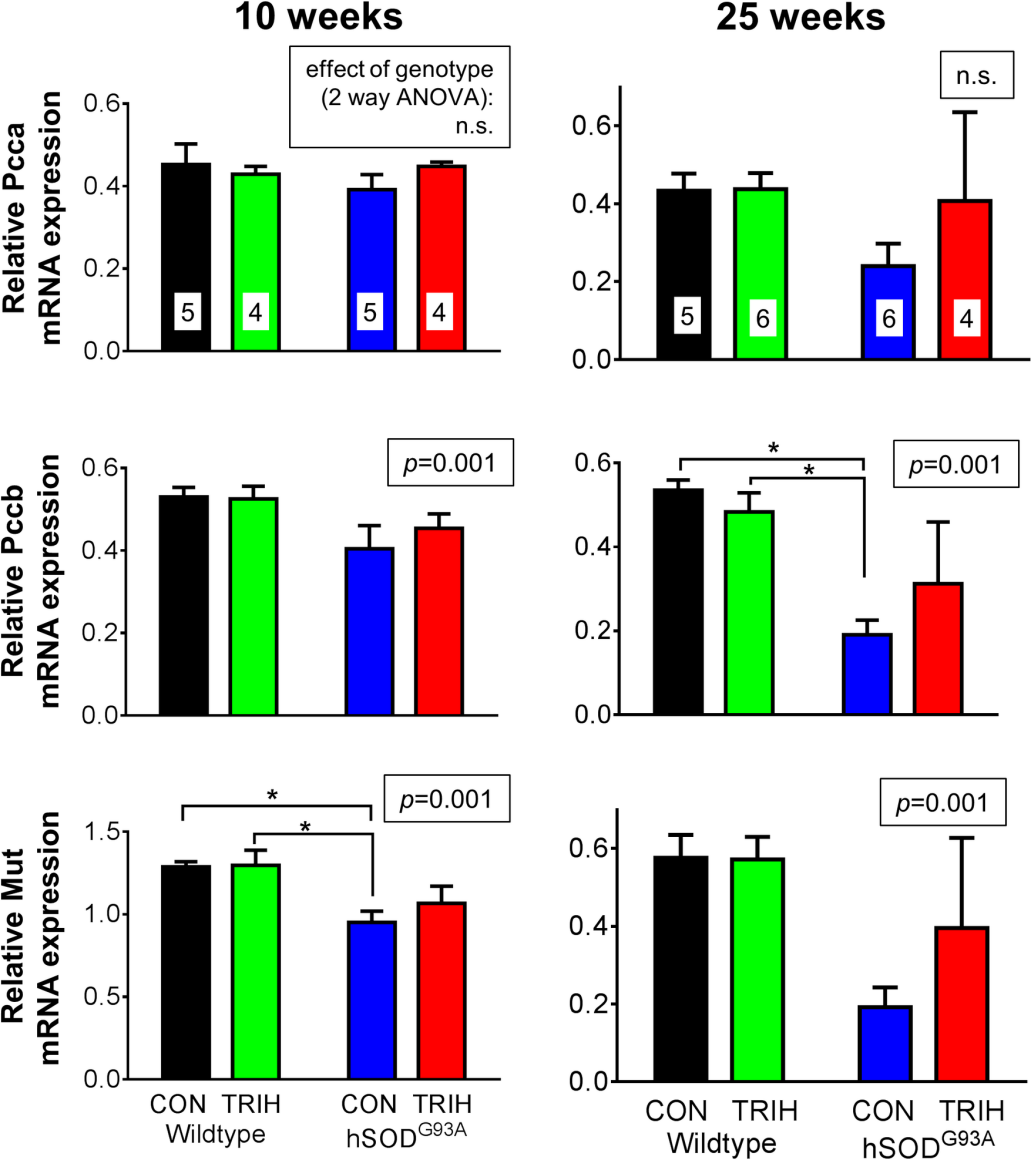
Gene expression of enzymes involved in the propionyl-CoA carboxylase pathway. Starting at P35, female wild-type and hSOD1^G93A^ mice were treated with triheptanoin (TRIH) or control (CON) diet until they were sacrificed. Relative expression of the α (*Pcca*) and β (*Pccb*) subunits of propionyl-CoA carboxylase and methylmalonyl mutase (*Mut*). Expresssion is compared in the gastrocnemius muscle of 10 and 25 week old wild-type and hSOD1^G93A^ mice untreated or treated with triheptanoin relative to housekeeping genes. N-numbers used for each group throughout the experiments are indicated in the top bar graphs. The insets above each graph show the p-values for the effects of genotype in two-way ANOVAs, while the effect of diet was p>0.05 for each bar graph. When significant, the results of Bonferroni post tests are indicated by a star (* p<0.05), indicating that triheptanoin treatment in hSOD1^G93A^ mice protected against lowered expression of *Pccb* and *Mut* mRNA.

Given the effect of triheptanoin on motor function in hSOD1^G93A^ mice, we next aimed to evaluate the extent to which TCA cycle metabolism or anaplerosis may be impaired in the gastrocnemius muscle of hSOD1^G93A^ mice in the early and late stages of the disease. We used quantitative real time PCR to compare the expression of genes encoding for enzymes involved in glycolysis (glyceraldehyde-3-phosphate dehydrogenase—*Gapdh*), the TCA cycle (pyruvate–*Pdha1*, 2-oxoglutarate—*Ogdh* and succinate dehydrogenases—*Sdha*, Fig 4) and anaplerotic pathways of the muscle. The latter include pyruvate carboxylase (*Pcx*) producing oxaloacetate, glutamic pyruvic transaminase 1 and 2 (G*pt1* and *2*, Fig 5), and the enzymes of the propionyl-CoA carboxylation pathway (Fig 6), namely the α and β subunits of propionyl-carboxylase (*Pcca* and *Pccb*) and methylmalonyl mutase (*Mut*), which together metabolize propionyl-CoA to succinyl-CoA. Triheptanoin treated mice were included in this analysis to investigate the effect of triheptanoin on the expression of these metabolic enzymes.

Investigating the effects of genotype and treatment on the expression of key metabolic enzymes, two way ANOVAs revealed that the genotype was linked to the variations observed in the expression of several enzymes relative to housekeeping genes at 10 and/or 25 weeks of age (p shown in insets of bar graphs, Figs 4–6, n = 4–6). When compared to wild-type mice, 10 week old hSOD1^G93A^ mice showed a reduction in the expression of several dehydrogenases and methyl-malonyl mutase; namely the mRNA levels of the A1 subunit of pyruvate dehydrogenase (*Pdha1*) were reduced by 24%, 2-oxoglutarate dehydrogenase (*Ogdh*) by 30%, the subunit A of succinate dehydrogenase (*Sdha*) by 23%, and methyl-malonyl mutase (*Mut*) by 27.5% in hSOD1^G93A^ mice (Figs 4 and 6; all p<0.05 in Bonferroni post-test, n = 4–6 mice per group). Triheptanoin attenuated the reduction in the expression of pyruvate and succinate dehydrogenases in hSOD1^G93A^ mice. However, triheptanoin had no effect on the expression of 2-oxoglutarate dehydrogenase and methylmalonyl mutase. No alterations of mRNA levels were evident in the other investigated genes, including glycolytic *Gapdh* and the genes involved in anaplerosis, *Pcx*, *Gpt1* and *2*, *Pcca* and *Pccb* (Figs 4–6).

At 25 weeks of age, the end-stage of disease [67,74], and relative to untreated wild-type mice, hSOD1^G93A^ mice showed reduced gene expression for succinate dehydrogenase (*Sdha*, 70%), *Gpt2* (84%) and the β subunit of propionyl carboxylase (*Pccb*, 64%) (all p<0.05 in post test, Figs 4–6). Triheptanoin prevented the reduction in the expression of these genes, indicating that it can preserve muscle energy metabolism.

**Fig 7:**
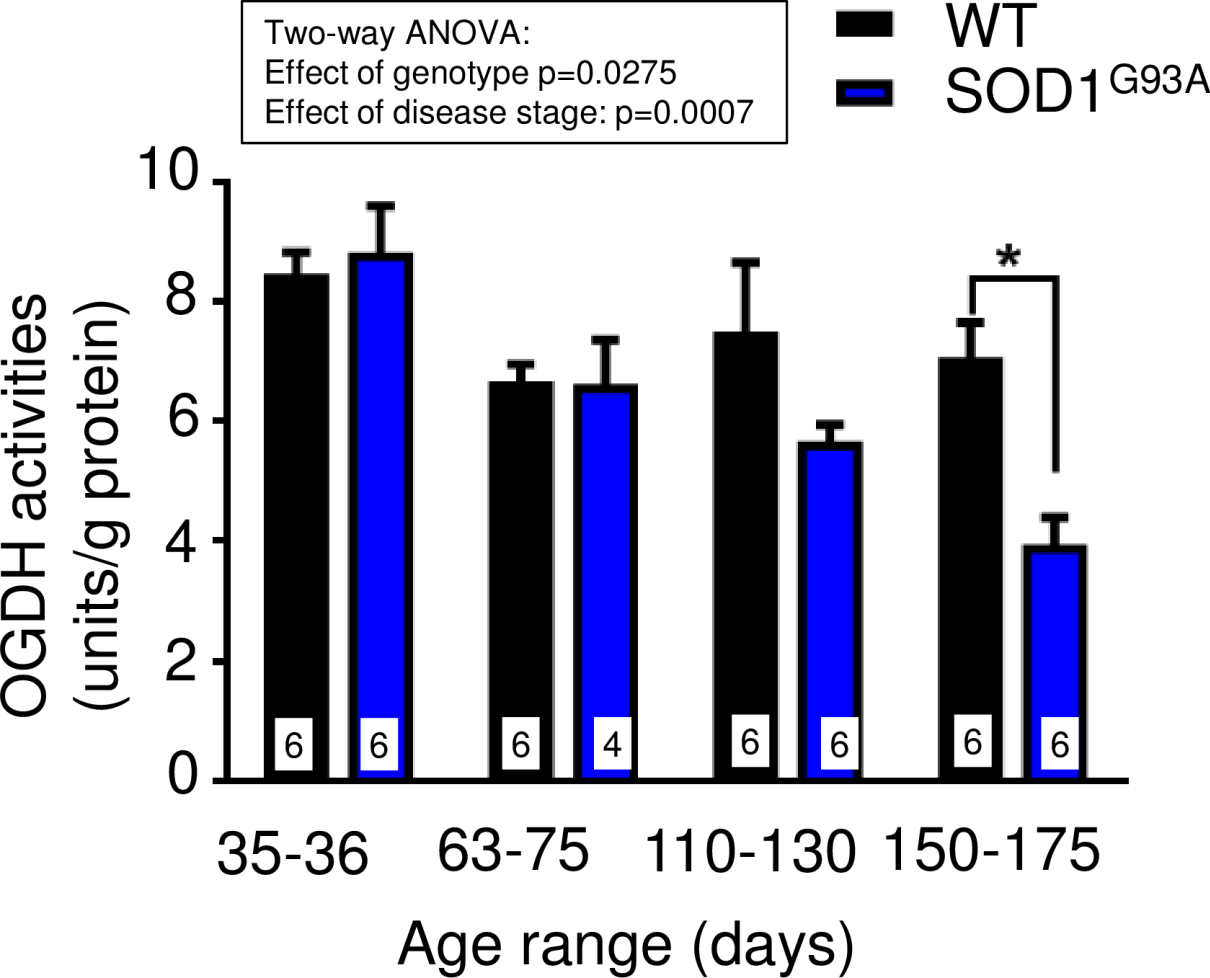
Lower maximal activities of 2-oxoglutarate dehydrogenase (OGDH) in hSOD1^G93A^ gastrocnemius muscle. The maximal activities of OGDH in extracts from gastrocnemius muscle of male wild-type and hSOD1^G93A^ mice at different disease stages are compared. Stages are defined as presymptomatic (days 35–36), onset (days 63–75), mid-stage (days 110–130) and end-stage (days 150–175). The inset above the graph shows the two-way ANOVA p-values for the effects of genotype and disease stage, indicating that 2-oxoglutarate activity in hSOD1^G93A^ mice declines with progression of disease. The star denotes significance in the Bonferroni post test (* p<0.05).

To investigate the extent to which these mRNA changes result in functional changes, we measured the maximal enzyme activity of 2-oxoglutarate dehydrogenase in extracts of gastrocnemius muscle in hSOD1^G93A^ compared to wild-type mice at different stages of disease (n = 4–6 mice each group). The two-way ANOVA analysis revealed that the maximal activity of this enzyme was significantly altered dependent on genotype (p<0.028) and disease stage (p<0.0007). At mid-stage (P110-130), there was a trend of a reduction by 25%, while the 45% loss of maximal 2-oxoglutarate dehydrogenase activity at end-stage (P150-175) was statistically significant by a Bonferroni multiple comparison post test (p<0.05, Fig 7).

### Triheptanoin slightly increased plasma ketone (β-hydroxybutyrate) levels

**Fig 8:**
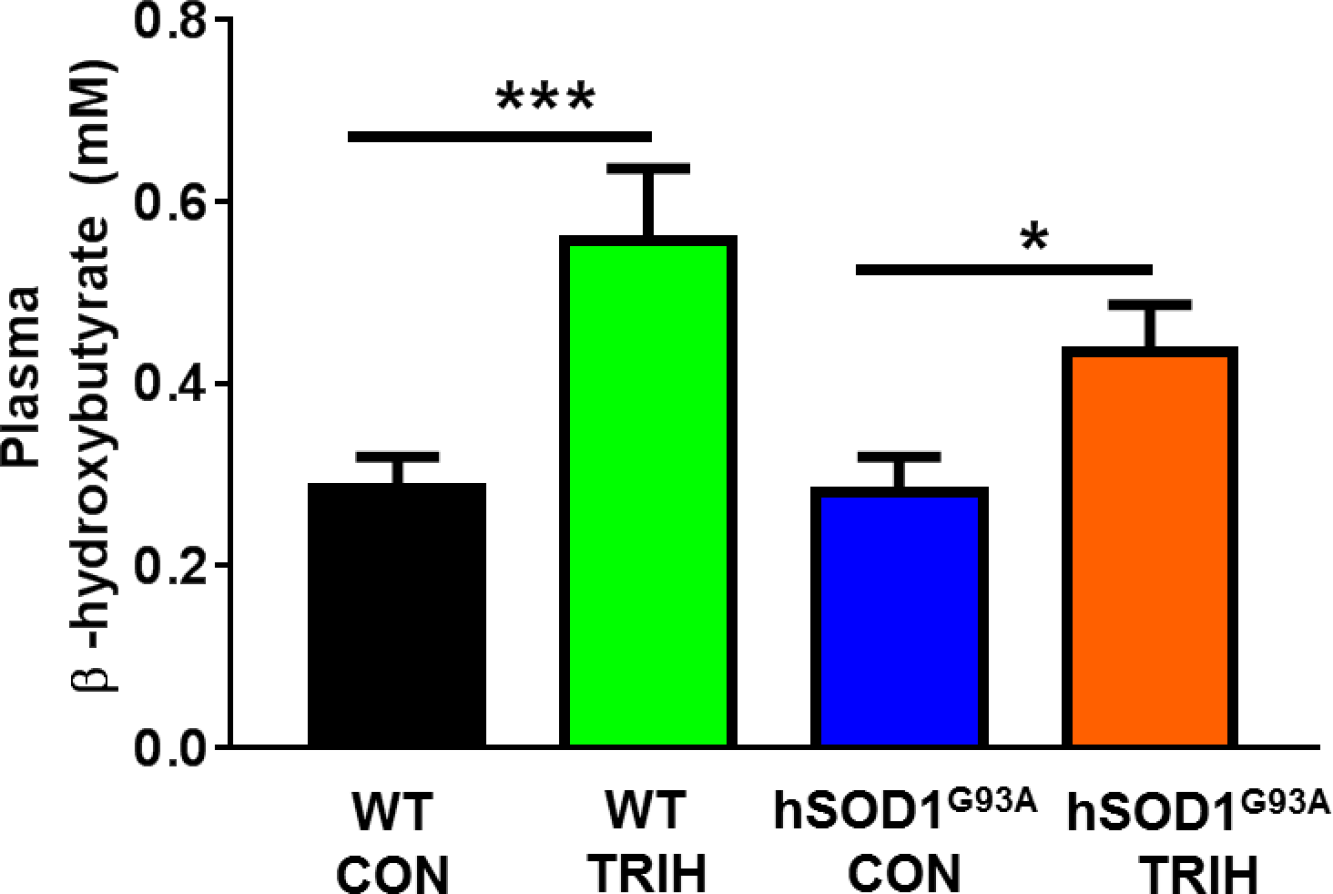
Triheptanoin treatment increased levels of plasma β-hydroxybutyrate. Plasma β-hydroxybutyrate levels (mM) in 70 days old wild-type and hSOD1^G93A^ mice fed with either triheptanoin (TRIH) or control (CON) diet from day 35 to 70 (One-way ANOVA p = 0.042; Fisher’s LSD post test: *p<0.05, ***p<0.001, n = 10–12).

Triheptanoin treated hSOD1^G93A^ mice showed a 56% increase in levels of plasma β-hydroxybutyrate compared to those treated with control diet (One-way ANOVA p = 0.042; p<0.05 Fisher’s LSD post test, Fig 8). Similarly, triheptanoin also increased plasma β-hydroxybutyrate levels in wild-type mice by 96% (p = 0.0008, p<0.001) (Fig 8). However, in both wild-type and hSOD1^G93A^ mice following triheptanoin treatment, no differences were seen in plasma glucose levels, with average levels between 213–219 mg/dl (One-way ANOVA, p = 0.99).

## Discussion

**Fig 9:**
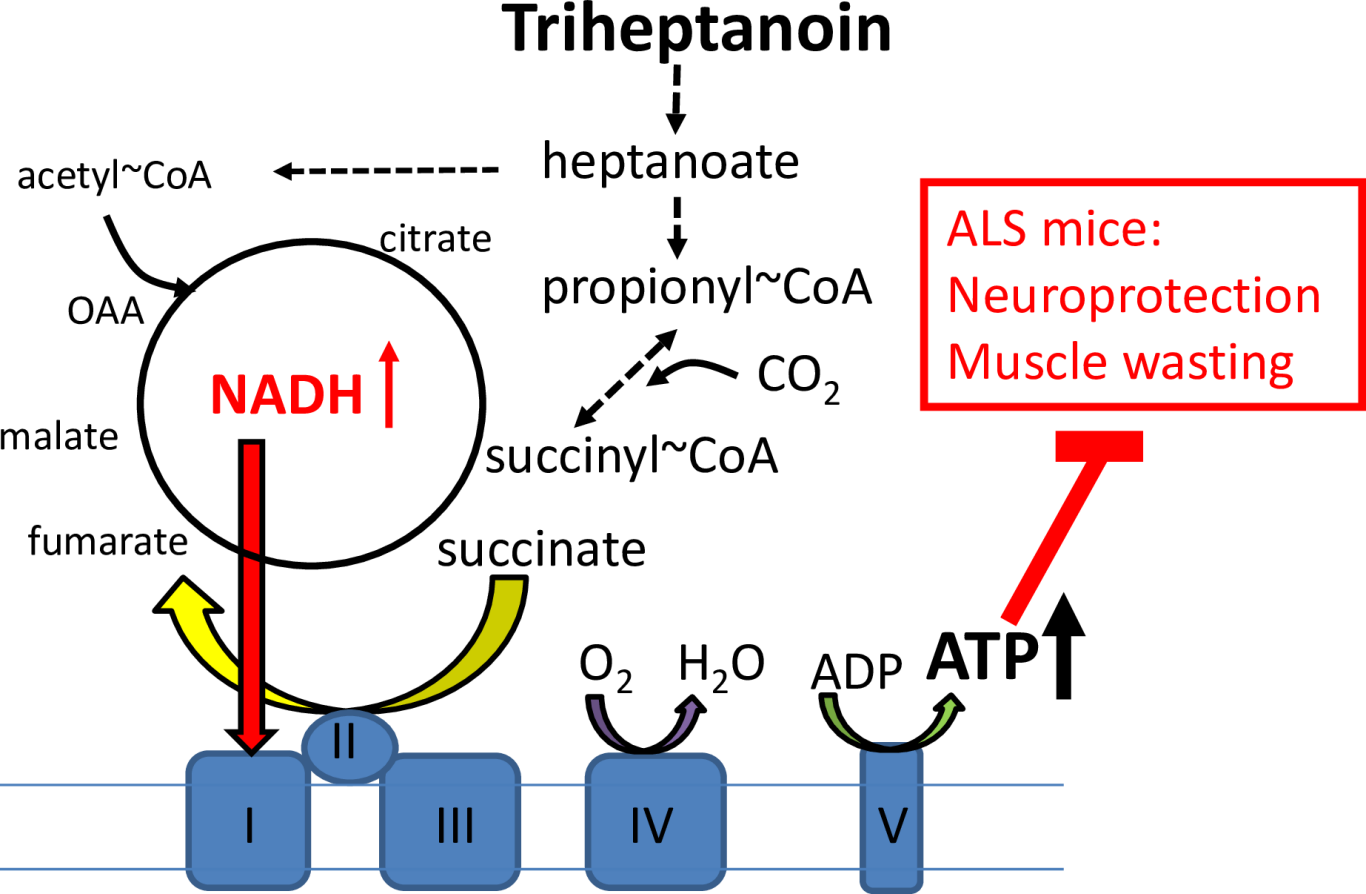
Hypothesized mechanisms of triheptanoin. Triheptanoin is the triglyceride of heptanoate, which is metabolized to acetyl-CoA as well as propionyl-CoA providing alternative and anaplerotic fuel. Following carboxylation propionyl-CoA produces succinyl-CoA (anaplerosis), which via metabolism to oxaloacetate can increase ATP production and aid in further acetyl-CoA oxidation. Thus triheptanoin can improve mitochondrial energy production and thereby protect neurons and muscle against degeneration.

The most important findings of this study are that 1) triheptanoin attenuated motor neuron loss in hSOD1^G93A^ mice, 2) triheptanoin delayed the onset of motor symptoms in hSOD1^G93A^ mice, and 3) decreased expression of muscle enzyme mRNA involved in TCA cycling in hSOD1^G93A^ mice was attenuated by triheptanoin. The hypothesized mechanisms by which triheptanoin protects motor neurons and delays muscle wasting is summarized in Fig 9.

### Neuroprotective Effects of Triheptanoin

In our study, triheptanoin treated hSOD1^G93A^ mice showed 33% less motor neuron loss in the L4-L5 spinal cord. Similarly, in two other neurological disorders models the same dose of triheptanoin was found to protect cells. In *nur7* mutant mice, a model for Canavan disease, triheptanoin treatment prevented the loss of oligodendrocytes [65]. In a mouse middle cerebral artery occlusion (MCAO) model of stroke, triheptanoin pre-treatment reduced the infarct area and mitochondrial function was preserved in mitochondria isolated from brains 1 h after onset of MCAO [64]. Based on the previous findings that triheptanoin diminished abnormalities in brain energy metabolism in different models and patients with CNS disorders [55,56,59,60] it is likely that improvements in ATP production contribute to triheptanoin’s neuroprotective effects. In addition, in an Alzheimer’s Disease model, triheptanoin in the context of a ketogenic diet increased the expression of the mRNA levels of *Sirt1*, *Pparg*, *Sod1* and *Sod2* [75]. Sirtuin 1 and PPAR-γ are regulators of lipid and glucose metabolism as well as mitochondrial respiration and oxidative stress. Thus, these studies indicate that the neuroprotective effects of triheptanoin may be mediated, in part, by limiting oxidative stress and by preserving mitochondrial function, which together with triheptanoin’s direct provision of alternative and anaplerotic fuel will contribute to improved energy supply.

### Clinical Importance of Triheptanoin’s Protective Effects on ALS Symptoms

In addition to its neuroprotective effects, triheptanoin delayed the onset of the loss of grip strength by 2.8 weeks in hSOD1^G93A^ mice. This corresponds to 11% of the life span and 30% of the “symptomatic” time in these mice. Thus, our data show an improvement in disease symptoms when treatment was initiated prior to the onset of disease symptoms in hSOD1^G93A^ mice. This raises hope that in ALS patients, triheptanoin may be able to preserve motor neurons and the function of muscles when treatment is initiated at an early stage of disease.

On the other hand, presymptomatic hSOD1^G93A^ mice already show loss of crural flexor motor neurons [67]. This indicates that in ALS mouse models, pathological neuron loss does not necessarily translate to discernable motor deficits in mice, while motor deficits in patients may be present at a similar pathological stage. Thus, while triheptanoin was given at a presymptomatic stage of disease in mice, it is difficult to correlate our findings to the clinical course of disease in patients. Given the difficulty in diagnosing ALS, future studies are required to evaluate the extent to which triheptanoin can preserve motor neurons and motor function when treatment is initiated in ALS models with obvious muscle and/or neuronal impairments.

As a medium chain triglyceride, triheptanoin quickly provides heptanoic acid and C5 ketones to the blood. These molecules cross the blood brain barrier and are expected to also be metabolized by muscle. Therefore, it is anticipated that the protective effects of triheptanoin will begin quickly after the initiation of treatment. Because triheptanoin was safe in pilot studies with patients with disorders involving severe metabolic deficiencies [54,76,77], CNS disorders [55,56,76], Pompe’s Disease and Inclusion Body Myositis [57], there are relatively few hurdles to overcome to start a phase I clinical trial to assess safety and tolerability for patients with ALS. It will be important to rule out potential and known metabolic contraindications in study subjects and to provide professional dietary advice in addition to triheptanoin treatment. To date, no central or cardiovascular side effects have been reported for triheptanoin. For medium chain fats, the most common side effects consist of abdominal pain, diarrhea and nausea, which can largely be controlled by mixing the treatment with food and slowly increasing the dose. Given previous reports of gastric dysfunction in ALS patients [78,79], it is also pertinent that the impact of triheptanoin on gut function in ALS be investigated.

As a means to circumvent potential side effects of oral triheptanoin on gut function, triheptanoin could be delivered intravenously in an emulsion. This route of delivery would circumvent the known side effects on the GI tract and supply heptanoate quickly to various tissues before it reaches the liver. The liver can convert heptanoate to glucose via gluconeogenesis and thus reduces the amounts of heptanoate and C5 ketones that can reach other tissues. Such a triheptanoin emulsion is currently being developed by industry. Triheptanoin is an ideal precursor to supply heptanoic acid and C5 ketones to the body, although these metabolites should also be directly effective for patients. However, large amounts of heptanoic acid and C5 ketones or their sodium salts are unsuitable for direct application, as they would add unphysiological levels of acid or salt to the body.

It is now thought that the majority of clinical trials addressing the efficacy of new treatments in ALS have failed due to the lack of solid preclinical data. Consequently, new guidelines have been developed for laboratory work before phase II trials [73], including power analyses and high animal numbers when claiming effects on survival. While our power analyses regarding loss of grip strength and balance show that our study used adequate numbers of mice regarding these analyses of motor symptoms, a larger and more complete preclinical study of triheptanoin following the mentioned guidelines would be ideal to test its effect on survival in multiple animal models of ALS. Also, treatment initiation during the symptomatic stage and assessments of muscle function and the integrity of neuromuscular junctions will be important to inform and motivate clinical trials.

### Metabolic Changes in hSOD1^G93A^ Mouse Muscle

When compared to healthy wild-type mice, we found that the expression of several enzyme mRNA involved in glycolysis, the TCA cycle and anaplerosis were significantly reduced in the gastrocnemius muscle of hSOD1^G93A^ mice at 10 weeks of age (Figs 4–6), a time when hind limb grip strength is still normal (Fig 3B). We also demonstrate that the maximal 2-oxoglutarate dehydrogenase activity was significantly reduced based on genotype and disease stage in gastrocnemius muscle in hSOD1^G93A^ compared to wild-type mice (Fig 7), indicating that the observed mRNA changes result in functional alterations. Taken together this suggests that ATP production is insufficient in the muscle for the function and maintenance of tissue at an early stage of the disease. Similar changes in mRNA levels were seen at the end-stage of disease at 25 weeks of age (Figs 4–6), highlighting that an impairment of TCA cycling may persist throughout the progression of disease.

Numerous studies have alluded to altered energy metabolism during the early and later stages of disease progression in muscle in ALS patients and in ALS mouse models [25,29–33,35]. In the majority of sporadic ALS patients, respiratory chain defects and multiple deletions in mitochondrial DNA have been observed in muscle biopsies [24], suggesting that metabolic abnormalities occur in muscle and that muscle pathology may contribute to ALS. Moreover, citrate synthase activity was decreased by 43% in muscle from ALS patients when compared to healthy controls [32]. Consistent with this, ALS patients displayed increased blood lactate levels, especially after exercise, indicating that acetyl-CoA is not metabolized well by the TCA cycle [80]. Furthermore, SOD1 mouse models of ALS showed reduced levels of ATP in skeletal muscle [35,36]. Moreover, oxidative stress, which is commonly observed in ALS (reviewed in [12,81]), is well known to diminish the activities of CNS and muscle aconitase and 2-oxoglutarate dehydrogenase, both enzymes of the TCA cycle [82,83].

At 25 weeks, we found reduced levels of the main anaplerotic enzyme mRNA of muscle, glutamic pyruvic transaminase 2, suggesting diminished levels of TCA cycle intermediates. Triheptanoin prevented the downregulation of several enzyme mRNA observed in hSOD1^G93A^ when compared to wild-type mice. This finding implies that normalization of energy metabolism by triheptanoin prevents downregulation of certain metabolism genes, which in turn would help to maintain a healthy metabolism to optimize energy supply, function and survival of tissue. When considering these collective metabolic alterations, it is not surprising that triheptanoin, which is currently the most effective treatment to produce anaplerotic propionyl-CoA slowed the onset of motor symptoms in hSOD1^G93A^ mice.

### Triheptanoin and Body Weight

Loss of body weight is a concern in many ALS patients and is an indicator for ALS progression during the later stages of disease in the hSOD1^G93A^ mouse. In this study, triheptanoin slowed body weight increases in the wild-type C57BL/6 mice. This effect has been previously mentioned for medium chain triglycerides, which in contrast to long chain fats, do not accumulate in adipose tissue (reviewed by [84]). However, in patients with muscular and neurological disorders, treatment with triheptanoin has not been reported to induce a loss in body weight. Rather increases in weight were seen in some patients [57]. Also, mice from two outbred strains gained weight equally with and without triheptanoin treatment [63,66]. In spite of a trend to lower body weight in this study, mice on triheptanoin showed similar hind limb grip strength when compared to untreated mice, indicating that lower body weight does not impair muscle function. In addition, despite this potential confounding factor, triheptanoin treated mice showed a delay in the loss of body weight and similar survival when compared to untreated mice. If triheptanoin treatment is tested in ALS models on background strains that do not exhibit a loss in body weight, it is likely that the disease modifying effects of triheptanoin will be further enhanced, potentially improving survival.

### Comparison to Other Metabolic Treatment Approaches for ALS

Other metabolic treatment approaches have been tested in hSOD1^G93A^ mice. Ketogenic therapy, which increases blood C4 ketone levels is commonly thought to improve the production of ATP by mitochondria [85]. When tested in hSOD1^G93A^ mice, a ketogenic diet preserved motor performance and delayed weight loss and loss of motor neurons in the lumbar spinal cord [86]. Similarly, trioctanoin (caprylic triglyceride, the triglyceride of octanoate), which also increases blood C4 ketone levels, increased oxygen consumption rates in mitochondria of the spinal cord, delayed onset of motor symptoms and protected motor neurons [87]. However, both treatments did not extend survival in hSOD1^G93A^ mice and ketogenic therapy is contraindicated in ALS as it typically leads to weight loss in adults. Our recent study indicated that chronic trioctanoin, but not triheptanoin, treatment can decrease glycolysis and the levels of TCA cycle intermediates in mouse brain tissue [88]. Thus, trioctanoin treatment alone might interfere with the generation of energy from glucose, which may worsen metabolic problems in ALS (reviewed in [89]). In the same study triheptanoin feeding to CD1 mice increased brain β-hydroxybutyrate levels 1.7-fold, which may contribute to its beneficial effects. Similarly, in the current study plasma levels of the C4 ketone β-hydroxybutyrate were increased with triheptanoin treatment in hSOD1^G93A^ mice by 1.6-fold (Fig 8). In addition, triheptanoin is expected to be superior to any fuel that produces C4 ketones, because triheptanoin is anaplerotic.

Dichloroacetate, a metabolic treatment that increases pyruvate dehydrogenase activity, has been found to improve survival time and/or delay the loss of motor function in SOD1 mice [49,90]. An increase in pyruvate dehydrogenase kinase activity is expected to allow more efficient production of energy from glucose, if there is sufficient TCA cycling. However, studies are needed to determine the impact of dichloroacetate on TCA cycle metabolism in order to allow a better understanding of this potential treatment approach in ALS.

There is evidence that some ALS patients benefitted from the “Deanna Protocol”, a complex combination of many metabolic supplements, in addition to medium chain triglyceride-rich coconut oil [91]. More recently, [92] showed the beneficial effects of a simplified “Deanna Protocol” in hSOD1^G93A^ mice. Specifically, a combination of arginine α-ketoglutarate, β-phenyl-γ-amino butyrate, ubiquinone and medium even chain triglyceride oil, which is largely a mixture of triglycerides of octanoate and decanoate, extended survival. Providing that α-ketoglutarate or a C5 or C4 metabolite of this compound enters mitochondria, the TCA cycle metabolite pool could be (re-)filled and this treatment can be considered as being “anaplerotic” similar to heptanoate. This would aid the metabolism of the co-supplied alternative medium chain fatty acids, octanoate and decanoate, which taken together closely resembles the proposed mechanism of action of triheptanoin. Despite current reports of beneficial outcomes in response to dietary intervention strategies in mouse models of ALS, the extent to which dichloroacetate, the Deanna protocol and triheptanoin are efficacious in controlled clinical trials remains to be investigated.

## Conclusion

This study revealed that triheptanoin is a promising new treatment approach for ALS to delay motor neuron loss and the onset of motor symptoms. Further studies with increased animal numbers and treatment initiation in symptomatic stages, and those that use ^13^C-labeled glucose or heptanoate are necessary to elucidate triheptanoin’s metabolic fate and mechanism of action in ALS. Such studies will be important to inform and optimize future clinical trials.
